# Fungal Glucosylceramide-Specific Camelid Single Domain Antibodies Are Characterized by Broad Spectrum Antifungal Activity

**DOI:** 10.3389/fmicb.2017.01059

**Published:** 2017-06-14

**Authors:** Barbara De Coninck, Peter Verheesen, Christine M. Vos, Inge Van Daele, Miguel F. De Bolle, Joao V. Vieira, Marnix Peferoen, Bruno P. A. Cammue, Karin Thevissen

**Affiliations:** ^1^Centre of Microbial and Plant Genetics, KU LeuvenLeuven, Belgium; ^2^Department of Plant Systems Biology, VIBGhent, Belgium; ^3^Agrosavfe NVGhent, Belgium

**Keywords:** camelid antibodies, VHH, antifungal activity, fungal glucosylceramide, *Botrytis cinerea*, crop protection

## Abstract

Chemical crop protection is widely used to control plant diseases. However, the adverse effects of pesticide use on human health and environment, resistance development and the impact of regulatory requirements on the crop protection market urges the agrochemical industry to explore innovative and alternative approaches. In that context, we demonstrate here the potential of camelid single domain antibodies (VHHs) generated against fungal glucosylceramides (fGlcCer), important pathogenicity factors. To this end, llamas were immunized with purified fGlcCer and a mixture of mycelium and spores of the fungus *Botrytis cinerea*, one of the most important plant pathogenic fungi. The llama immune repertoire was subsequently cloned in a phage display vector to generate a library with a diversity of at least 10^8^ different clones. This library was incubated with fGlcCer to identify phages that bind to fGlcCer, and VHHs that specifically bound fGlcCer but not mammalian or plant-derived GlcCer were selected. They were shown to inhibit the growth of *B. cinerea in vitro*, with VHH 41D01 having the highest antifungal activity. Moreover, VHH 41D01 could reduce disease symptoms induced by *B. cinerea* when sprayed on tomato leaves. Based on all these data, anti-fGlcCer VHHs show the potential to be used as an alternative approach to combat fungal plant diseases.

## Introduction

Application of fungicides has been indispensable in modern agriculture to control fungal diseases and ensure sufficient crop yields. However, such chemical crop protection products face many challenges including the emergence of resistant pathogens and the search for new active compounds that combine high activity with favorable toxicology profiles. Therefore, research is increasingly focusing on innovative and flexible solutions to provide more efficient crop protection.

In nature, plants defend themselves against fungal pathogens by a combination of strategies including the production of antimicrobial peptides and proteins. An important and evolutionary conserved family of such antimicrobial peptides are the plant defensins (Terras et al., 1995). They are small, cationic peptides with a length of approximately 45–54 amino acids. Their structure typically comprises a cysteine-stabilized αβ-motif (CSαβ) with a α-helix and a triple-stranded antiparallel β-sheet that is stabilized by four disulfide bridges. Their activity is primarily directed against fungi, but bactericidal and insecticidal actions have also been reported (De Coninck et al., 2013). The mode of action of various antifungal plant defensins has been extensively studied during the last decades and several of their fungal targets have been identified to date (Vriens et al., 2014). Different plant defensins specifically interact with different classes of sphingolipids in the fungal membrane, either inositolphosphoryl-containing sphingolipids or glucosylceramides (GlcCer). With respect to the latter, plant defensins of radish (RsAFP2; Thevissen et al., 2004), pea (Psd1; de Medeiros et al., 2010; Gonçalves et al., 2012) and alfalfa (MsDef1; Ramamoorthy et al., 2007) as well as an insect defensin of tobacco budworm (heliomicin; Thevissen et al., 2004) specifically interact with fungal GlcCer (fGlcCer). GlcCer are ubiquitous eukaryotic membrane components that have structural as well as signaling functions in eukaryotes (Thevissen et al., 2007). Apart from GlcCer-interacting defensins, there are indications that also antibodies directed toward fGlcCer possess antifungal activity (Nimrichter et al., 2004). Hence, it seems that various proteins or peptides that interact with fGlcCer can inhibit fungal growth to some extent. This makes fGlcCer an attractive target for development of novel antifungal compounds for several reasons (Thevissen et al., 2005; Nimrichter and Rodrigues, 2011; Del Poeta et al., 2014). First, the structure of fGlcCer is highly conserved in a broad spectrum of fungi while fGlcCer are structurally different from their mammalian or plant counterparts (Thevissen et al., 2007; Nimrichter and Rodrigues, 2011). This host-specificity ensures non-toxicity of compounds that target fGlcCer while maintaining a broad spectrum of antifungal activity. Second, fGlcCer are important virulence factors of pathogenic yeasts and fungi (Noble et al., 2010). Therefore, it can be expected that *in vivo* resistance development against the GlcCer-targeting compounds is unlikely to occur. Indeed, an altered GlcCer composition of the target pathogen will result in resistance against the GlcCer-targeting compounds, but concomitantly also in reduced pathogenicity of the mutant pathogen.

The general aim of this study was to generate novel compounds that interact with fGlcCer and are characterized by antifungal activity. To this end, we relied on the antigen-binding domain of camelid heavy chain antibodies. Camelid species have, in addition to conventional IgG antibodies, heavy chain antibodies that are composed of homodimers of two heavy chains only (Hamers-Casterman et al., 1993). Conventional IgG antibodies consist of three major fragments: two identical antigen-binding fragments and an Fc part (Padlan, 1994). The antigen-binding fragment in conventional antibodies (Fab) consists of a light chain of two domains (V_L_-C_L_) and two of four domains of the heavy chain (V_H_-C_H_1). Functional antigen binding of heavy chain antibodies is achieved by a single domain that is called VHH, a small 15 kDa protein with excellent stability and particularly high affinity and specificity for the targets to which they are raised. VHHs can be easily cloned as they are encoded by single genes, have high antigen affinity and selectivity, possess good solubility and can be efficiently produced in micro-organisms such as *Escherichia coli*, *Saccharomyces cerevisiae*, or *Pichia pastoris* (Arbabi Ghahroudi et al., 1997; Frenken et al., 2000; van de Laar et al., 2007).

Hence, in this study, we engineered fGlcCer-targeting VHHs and assessed their GlcCer-interacting capacity and antifungal activity *in vitro* and *in vivo* against plant pathogenic fungi.

## Materials and Methods

### Materials

Purified (99%) glucosylceramide (GlcCer) from *Pleurotus citrinopileatus* (Tamigotake) was purchased form Nacalai Tesque (Japan). Soybean and porcine GlcCer were purchased from Avanti Polar Lipids (Alabama, United States). *Fusarium oxysporum* GlcCer was obtained from Professor Eliana Barreto Bergter of the Universidade Federal do Rio de Janeiro (Brazil). For immunization, GlcCer from *P. citrinopileatus* was dissolved in a methanol:chloroform:water mixture (16:16:5, v/v/v) and spotted on a TLC (Thin Layer Chromatography) silica glass plate (Sigma–Aldrich). Silica with adsorbed GlcCer was scraped from the plate and suspended in phosphate buffer (1 mg/ml). The other GlcCer samples were dissolved in a chloroform:methanol mixture (2:1, v/v).

For immunization, a mixture was prepared from 1E+07 *Botrytis cinerea* (B05.10) spores (see below) and 3.5 mg homogenized *B. cinerea* mycelium. For the latter, *B. cinerea* mycelium was grown in culture flasks containing half strength PDB (12 g/l Potato Dextrose Broth medium (Lab M, United Kingdom)) for 3 days at room temperature. The mycelium was recovered using a filter covered with Miracloth and resuspended in phosphate buffer.

*Botrytis cinerea* (R16, B05.10, kindly provided by Rudi Aerts, KU Leuven, Belgium), *Verticillium dahliae* (MUCL19210), *F. culmorum* (MUCL30162), *F. graminearum* (MUCL30161) and *Alternaria brassicicola* (MUCL20297) were used to test the antifungal activity of VHHs. Cultivation and spore harvesting was performed as described previously (Broekaert et al., 1990). Spores were collected from *V. dahliae* and *B. cinerea* grown on half strength PDB (12 g/l Potato Dextrose Broth medium (LABM, United Kingdom), 15 g/l select agar) and *A. brassicicola, F. culmorum*, and *F. graminearum* on 6CA-medium (cereal agar; 20 g/l baby cereals (Nestlé), 15 g/l select agar).

Recombinant *Raphanus sativus* AFP2 (RsAFP2) was produced in *P. pastoris* and purified from the supernatant as previously described (Vriens et al., 2016).

Tomato (*Solanum lycopersicum*) cv. Castlemart was grown at 22°C in a greenhouse with 75% relative humidity in a 16-h light/8-h dark regime.

### Animal Immunizations

Two llamas were subcutaneously immunized twice with 100 μg GlcCer and four times with 50 μg GlcCer together with incomplete Freund’s adjuvant (Sigma–Aldrich) following a two-weekly time schedule. Subsequently these llamas were also boosted three times with a mixture of 1E+07 *B. cinerea* spores and 3.5 mg mycelium following a two-weekly time schedule. All llamas remained healthy throughout the immunization process and blood samples were taken 7 days after the last GlcCer immunizations and *B. cinerea* boost, respectively. All vaccination experiments are executed according to EU animal welfare legislation and after approval of the local ethics (CNREEA : C2EA – 14). All animals are registered, manipulated by authorized staff, and an experienced veterinarian.

### Library Construction

**FIGURE 1:**
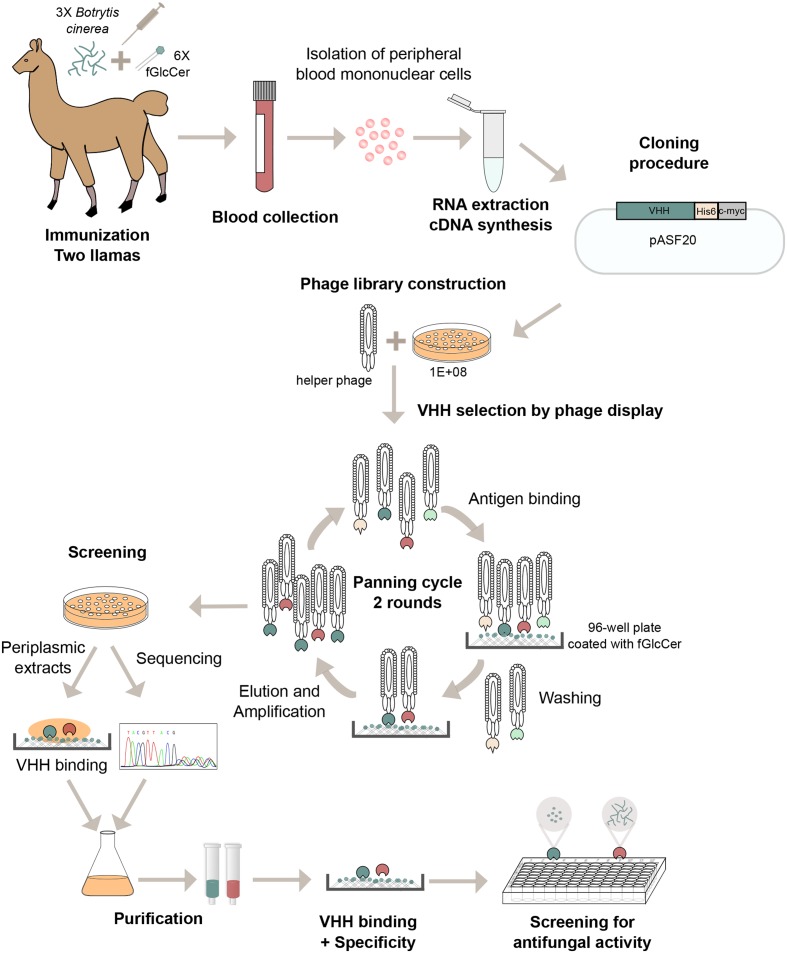
Schematic overview of library construction and screening of VHHs with fGlcCer binding and antifungal activity.

**Figure 1** gives a schematic overview of the library construction. Peripheral blood mononuclear cells were prepared from 0.4 L blood of the immunized llamas using Ficoll-Hypaque according to the manufacturer’s instructions (GE Healthcare). Total RNA was extracted from these cells using the RNeasy Maxi Kit (Qiagen) and used as starting material for cDNA synthesis using the Superscript III First-strand cDNA kit (Invitrogen). First strand cDNA synthesis is followed by RT-PCR using the forward primers Lib1 (5′-GGCTGAGCTGGGTGGTCCTGG-3′) and Lib2 (5′-GGCTGAGTTTGGTGGTCCTGG-3′) and the reverse primer Lib3 (5′-GGTACGTGCTGTTGAACTGTTCC-3′) (de Haard et al., 2014). The VHH encoding gene fragments are excised from agarose gel and amplified by PCR using the following specific primers: Lib5 (5′-AAATGAGGAGACGGTGACCTGGGT) and Lib7 (5′-CATTTGAGTTGGCCTAGCCGGCCATGGCACAGGTGCAGCTGCAGGAGTCTGGGGG-3′). The gel-purified PCR-products were digested with SfiI and Eco91I and ligated into the phagemid vector pASF20. pASF20 is an expression vector that is derived from pUC119 which contains the lacZ promotor, a synthetic leader sequence, a multiple cloning site, a coliphage pIII protein coding sequence, a resistance gene for ampicillin, and an M13 phage origin for single strand production. In frame with the VHH coding sequence, the vector codes for a C-terminal (His)6 peptide tag and c-myc peptide tag. The ligation products were transformed into TG1 *E. coli* competent cells. Four libraries each with a clonal diversity equal to or greater than 1E+08 were obtained.

### VHH Selection by Phage Display

Phages were prepared according to standard methods described in Kay et al. (1996) and phage display selection was performed according to Verheesen and Laeremans (2012).

Fungal glucosylceramides from *P. citrinopileatus* and *F. oxysporum* were used as targets to isolate the phage expressing fGlcCer-binding VHHs. fGlcCer from *P. citrinopileatus*, dissolved in methanol:chloroform:water mixture (16:16:5, v/v/v) and the fGlcCer from *F. oxysporum* dissolved in chloroform:methanol (2:1, v/v) were diluted in methanol and immobilized on polystyrene Nunc Maxisorp^®^ 96-well plates by overnight evaporation at room temperature. Wells with coated fGlcCer (10 μg for *P. citrinopileatus* GlcCer and 1 μg for *F. oxysporum* GlcCer in 100 μl) were washed three times with phosphate buffer and blocked with 1% fish gelatin (Sigma–Aldrich). Aliquots (100 μl) of the blocked phage premix were added to the wells and incubated for 2 h at room temperature on a shaking platform. The wells were washed 15 times with phosphate buffer and bound phages were eluted with trypsin (0.1 mg/ml). Two consecutive rounds of selections were performed, one campaign using *P. citrinopileatus* GlcCer in the first round and *F. oxysporum* GlcCer in the second round and one campaign using *F. oxysporum* GlcCer in both rounds. Individual clones were picked from second round selections for further characterization by sequence analysis (LGC group) and primary binding assays. Periplasmic extracts were prepared from 96-well plate produced cultures. Nunc Maxisorp^®^ plates were coated with 1 μg *F. oxysporum* GlcCer as described above. The plates were blocked with 1% fish gelatin and 10-fold diluted VHH-containing periplasmic extracts were incubated for 1 h at room temperature in 1% BSA/1% Gelatin. After the plates were washed they were incubated with mouse anti-histidine antibodies and anti-mouse alkaline phosphatase antibodies (Sigma–Aldrich). Finally, the enzyme activity was determined with the alkaline phosphatase substrate (Sigma–Aldrich). The optical density was measured at 405 nm.

### VHH Purification

For further characterization, VHHs were produced in *E. coli* in culture flasks according to standard procedures (Marks et al., 1991). His-tagged VHHs were purified from the periplasmic extracts using the ÄKTAxpress using a combination of immobilized Nickel IMAC and desalting columns, according to the manufacturer’s instructions (GE Healthcare Life Sciences).

### Characterization of Anti-GlcCer VHH Using ELISA

Nunc Maxisorp^®^ plates were coated with 1 μg *F. oxysporum*, 10 or 1 μg *P. citrinopileatus* and 1 μg soybean or porcine GlcCer as described above. The plates were blocked with 1% fish gelatin and purified VHHs (1 μg/ml) were incubated for 1 h at room temperature in 1% BSA/1% Gelatin. After the plates were washed they were incubated with mouse anti-histidine antibodies and anti-mouse alkaline phosphatase antibodies (Sigma–Aldrich). Finally, the enzyme activity was determined with the alkaline phosphatase substrate (Sigma–Aldrich). The optical density was measured at 405 nm.

### Antifungal Activity

The antifungal activity of the VHHs against a range of plant pathogenic fungi was analyzed following the protocol previously described (Osborn et al., 1995) with some modifications. Briefly, a twofold dilution series of the VHHs in sterile water was prepared in 96-well plates, after which 10 μl of VHH was mixed with 90 μl of half strength PDB containing 5E+04 spores/ml of the fungus. 96-well plates were incubated at 21°C in the dark for 48 or 72 h after which the mycelium growth was visually determined as compared to the control (i.e., water) treatment, using an inverted microscope (Motic AE21).

### Disease Assay

Detached leaves of 6-week old tomato plants were sprayed using an airbrush (VidaXL, nozzle diameter of 0.35 mm) until runoff with 60 μM VHH 41D01 resuspended in 0.2 × PBS (phosphate buffered saline) Tween20 (0.01%) or with a mock solution [0.2 × PBS Tween20 (0.01%)]. After 24 h, 10 leaves of five different plants were each inoculated with four 5-μl droplets of 1E+05 spores/ml of *B. cinerea* B05.10 and stored in plastic chambers on wet paper at high humidity (>99%). Lesion diameters were measured 3, 4, and 5 days post inoculation. Data were statistically analyzed with Two-way ANOVA and Bonferroni post-test. The experiment was repeated three times with similar results.

## Results

### Generation and Selection of fGlcCer-Targeting VHHs

**FIGURE 2:**
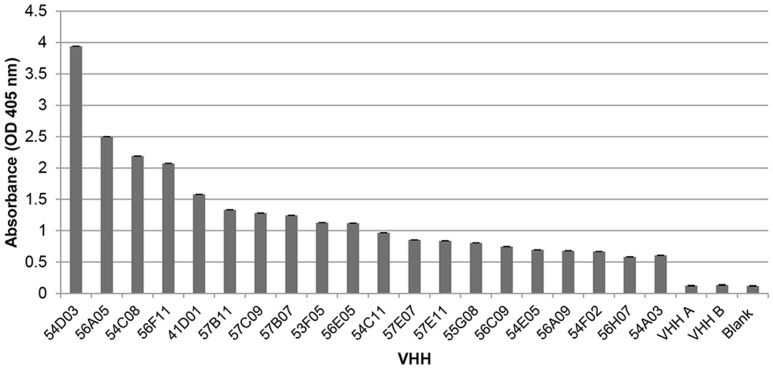
Interaction of VHHs with fGlcCer. Optical density values (absorbance at 405 nm) represent the interaction of 10-fold diluted crude VHH-containing periplasmic extracts to 10 μg coated GlcCer from *Pleurotus citrinopileatus*. VHH A and B are unrelated VHHs; the blank control represents a value for the ELISA experiment without VHH present.

The aim of this study was to generate VHHs against fGlcCer and to select those VHHs that exhibit binding to fGlcCer. **Figure 1** provides a schematic overview of the library construction. To select for fGlcCer-binding VHHs via phage display, two llamas were immunized with six boosts of commercially available fGlcCer from the fungus *P. citrinopileatus* and three additional boosts of a mixture of homogenized mycelium and spores of the fungus *B. cinerea*. All llamas remained healthy throughout the immunization process. Four phage libraries each with a clonal diversity higher than 1E+08 were obtained and used in the selection procedure (**Table 1**). For this, fGlcCer from *P. citrinopileatus* or *F. oxysporum* were coated and incubated with phages. Non-binding and fast-dissociating phages were washed away and specific phages remained bound to the fGlcCer. After two rounds of selections a total of 12 master plates containing 1128 individual clones were selected for further analysis by sequencing and primary binding assays (**Table 1**). Primary binding assays were performed via an ELISA-based binding assay with VHH-containing periplasmic extracts and GlcCer from *P. citrinopileatus*. Interestingly, 130 VHH-containing periplasmic extracts showed to bind fGlcCer with higher OD 405 nm values than the unrelated VHH A and VHH B or blank controls (**Supplementary Figure S1**), indicating that the enrichment was successful. Sequence analysis revealed 79 unique sequences from the identified set of anti-GlcCer VHH. To select the strongest binding and most specific fGlcCer interactors for further study we also took into account the binding ratio - i.e., the ratio of the absorbance of the interaction experiment with and without fGlcCer. Clones with the highest ratio were selected. **Figure 2** represents the 20 most potent VHHs with a binding ratio higher than 4.

**Table 1:** Overview of immunizations, libraries and master plate selections.

Immunization	Boost	Library sets	Selection	Master plates
GlcCer from *P. citrinopileatus*	*B. cinerea* spores and mycelium	Four libraries (2 after *P. citrinopileatus and 2 after P. citrinopileatus* + *B. cinerea* boost)	1st round with GlcCer from *P. citrinopileatus*	MP39 – MP40


			1st round with GlcCer from *P. citrinopileatus* and 2nd round with GlcCer from *F. oxysporum*	MP41 – MP42 MP50 – MP53
			Two rounds with GlcCer from *F. oxysporum*	MP54 – MP57


**FIGURE 3:**
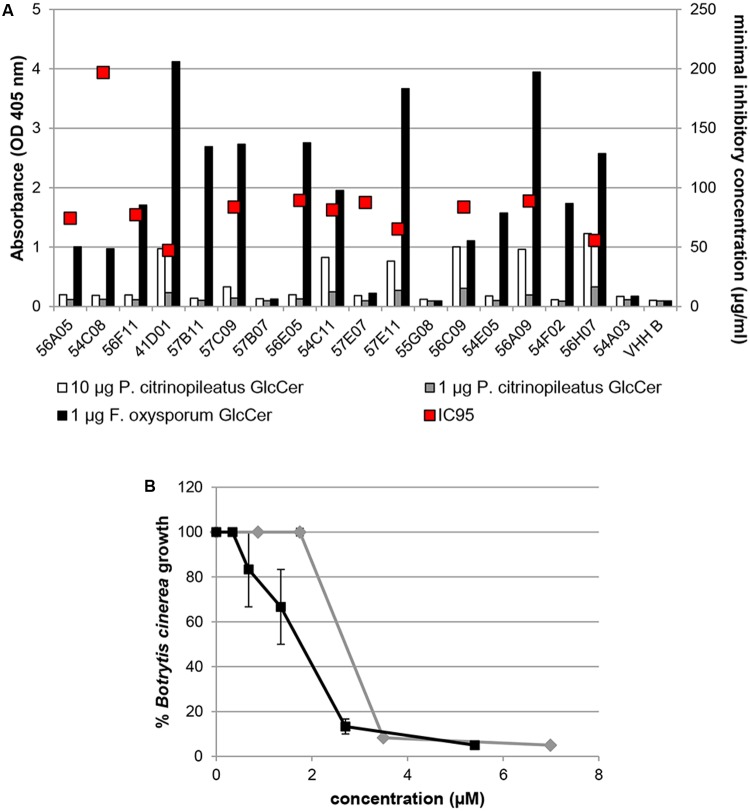
Specific Interaction of VHHs with fGlCer and antimicrobial activity of selected VHHs. **(A)** Optical density values (absorbance at 405 nm, depicted in left *Y*-axis) represent the interaction of 1 μg/ml purified VHH to coated GlcCer from *Fusarium oxysporum* or *Pleurotus citrinopileatus* and was evaluated in an ELISA-based binding assay. VHH B is an unrelated VHH. The antimicrobial activity of 12 VHHs was tested against *Botrytis cinerea.* Growth of *B. cinerea* R16 mycelium was microscopically determined 48 h after addition of different concentrations of VHHs. Minimal inhibitory concentrations at which at least 95% of the growth of the fungus was inhibited (IC_95_), compared to the control without added VHH, are indicated with a red dot and represent an average of two biological replicates. IC_95_ values are depicted in the right *Y*-axis. **(B)** Percentage growth of *B. cinerea* 48 h post co-incubation with different concentrations of VHH 41D01 (gray line) or the plant defensin RsAFP2 (black line). Data points represent the mean ± SE of three biological replicates.

To further characterize the selected VHHs, a specificity ELISA was performed. For this, the selected VHHs were produced in *E. coli* and purified from the periplasmic extracts. Typical yields were 0.5–10 mg purified VHH per liter culture. VHHs 54D03 and 53F05 were not obtained in sufficient quantities and therefore not pursued further. To test binding specificity of the purified VHHs, 1 μg/ml VHH was incubated with coated GlcCer from *P. citrinopileatus*, *F. oxysporum*, *Glycine max* (soybean) and porcine. Although the binding capacity of the VHHs toward the fGlcCer varied strongly (**Figure 3A**), none of the VHHs showed binding capacity toward plant or mammalian GlcCer (**Supplementary Figure S2**).

### fGlcCer-Binding VHHs Display *In Vitro* Antifungal Activity

**FIGURE 4:**
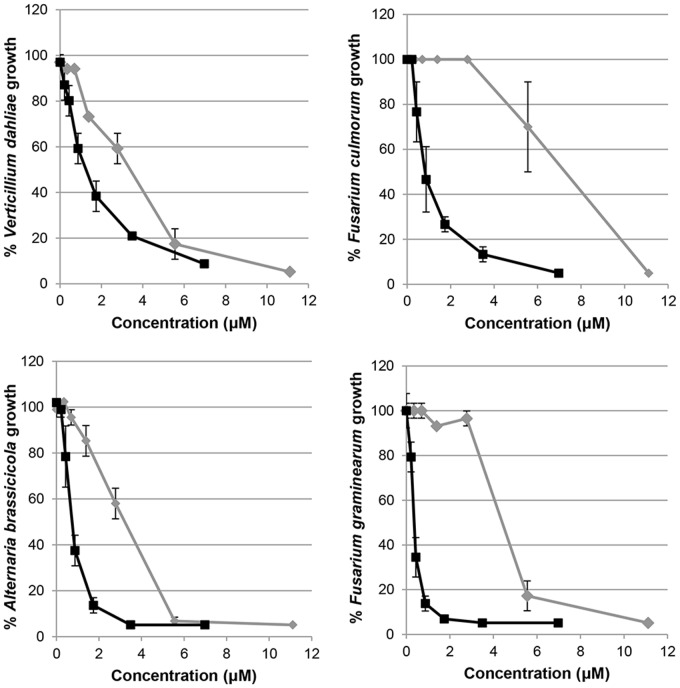
Antifungal activity of VHH 41D01. Graphs represent percentage growth of the different fungi *Verticillium dahliae* (96h post incubation, hpi), *F. culmorum* (72hpi), *Alternaria brassicicola* (72hpi) and *F. graminearum* (96hpi) post co-incubation with different concentrations of RsAFP2 (black lines) and VHH 41D01 (gray lines). Data points represent the mean ± SE of three biological replicates.

Twelve VHHs were selected for further characterization and tested *in vitro* for antifungal activity against *B. cinerea.* The minimal inhibitory concentration at which 95% of the fungal growth was inhibited (IC_95_) varied between 47 and 198 μg/ml (between 3 and 13 μM) (**Figure 3A**) with VHH 41D01 having the highest activity, and approximately as potent as the plant defensin RsAFP2 (**Figure 3B**). To test whether VHH 41D01 has broad antifungal activity we determined its inhibitory activity against the fungal plant pathogens *F. culmorum*, *F. graminearum*, *V. dahliae*, and *A. brassicicola.* VHH 41D01 also inhibited the growth of these fungi (IC_95_ < 12 μM) (**Figure 4**) showing it has broad spectrum activity. RsAFP2 was taken along as positive control.

### VHH 41D01 Reduces Disease Symptoms of *B. cinerea* When Sprayed on Tomato Leaves

**FIGURE 5:**
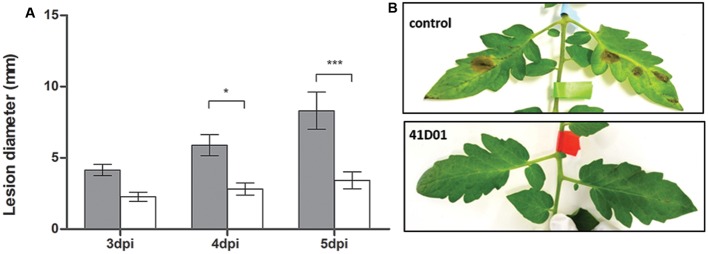
*In vivo* antifungal activity of VHH 41D01. Tomato leaves were sprayed with VHH 41D01 or a control solution [0.2 × PBS Tween20 (0.01%)]. After a drying period of 24 h, leaves were inoculated with 5 μl drops of 1E+05 spores/ml of *B. cinerea*. **(A)** Bars represent average lesion diameter (mm) scored at 3, 4, and 5 days post inoculation (dpi), error bars represent standard errors of the mean with *n* = 10. Significant differences between control treatment (gray bars) and VHH 41D01 treatment (white bars) are marked with an asterisk (^∗^*p* < 0.05, ^∗∗∗^*p* < 0.001). **(B)** Picture of tomato leaves treated with VHH 41D01 showing no infection after artificial inoculation compared to leaves treated with mock solutions.

To further test the *in vivo* efficacy of VHH 41D01, we evaluated disease symptom development of detached tomato leaves inoculated with *B. cinerea* (B05.10) 24 h after spraying the leaves with 60 μM VHH 41D01 or a mock solution [0.2 × PBS Tween20 (0.01%)]. Compared to the control treatment, VHH 41D01 reduced disease symptoms induced by *B. cinerea* by approximately 50% (**Figure 5**).

## Discussion

Here we show that anti-fGlcCer VHHs have potential to combat fungal plant diseases. Previously, several reports focusing on human fungal pathogens showed that fGlcCer is a potential target for the development of novel antifungals (Thevissen et al., 2005; Nimrichter and Rodrigues, 2011; Del Poeta et al., 2014). First, GlcCer are ubiquitous eukaryotic membrane components, yet those found in fungal cells are structurally different from those in human cells. Indeed, we confirmed that anti-fGlcCer VHHs specifically bind fGlcCer without cross-reactivity for mammalian or plant GlcCer (**Supplementary Figure S2**). Secondly, fGlcCer are important for fungal development including cell division, hyphal formation, alkaline tolerance and spore germination (Nimrichter and Rodrigues, 2011; Del Poeta et al., 2014). In addition, fGlcCer appear to play a central role in signal transduction and cell regulation (Hannun and Luberto, 2000; Thevissen et al., 2006). Thirdly, GlcCer of human fungal pathogens are correlated with fungal pathogenicity (Luberto et al., 2001; Rittershaus et al., 2006; Noble et al., 2010; Nimrichter and Rodrigues, 2011). For example, a Δ*gcs1* strain of *Cryptococcus neoformans*, lacking the GlcCer synthase (GCS), loses its pathogenicity. Also for fungal plant pathogens GlcCer seem to play a role in pathogenicity although this effect seems to depend on the host. While a Δ*gcs1* strain from the fungal plant pathogen *F. graminearum* is less virulent on wheat spikelets it retains full virulence on tomato fruits and *Arabidopsis thaliana* floral and foliar tissues (Ramamoorthy et al., 2007). In accordance, a Δ*gcs1* strain of *Penicillium digitatum* showed reduced symptom development on citrus fruits compared to the wild-type strain (Zhu et al., 2014).

Antifungal compounds that either directly target fGlcCer or its biosynthesis and, hence, affect fungal development and infection, can be expected to combat efficiently fungal infections. Indeed, several antimicrobial plant defensins and the insect defensin heliomicin, have been reported to interact specifically with fGlcCer or require fGlcCer to exert their antifungal activity (Thevissen et al., 2004; Ramamoorthy et al., 2007; de Medeiros et al., 2010). More specifically for plant pathogenic fungi, it has been reported that the Δ*gcs1* strain of *F. graminearum* is resistant to defensins from, e.g., *Medicago sativa* (MsDef1) and radish (RsAFP2) (Ramamoorthy et al., 2007). Moreover, transgenic expression of both plant defensins in various crop plants rendered the plants less susceptible to fungal pathogens (De Coninck et al., 2013). In line, anti-GlcCer antibodies that specifically interact with fGlcCer seem to inhibit fungal growth *in vitro* and also *in vivo* since it has been demonstrated that passive immunization with a monoclonal antibody to GlcCer significantly reduces host inflammation and prolongs the survival of mice lethally infected with *C. neoformans* (Rodrigues et al., 2000, 2007; Nimrichter et al., 2004). Also inhibitors of fGlcCer were reported to be highly effective *in vitro* and *in vivo* against fungal human pathogens (Mor et al., 2015). Recently, it was shown that administration of fGlcCer in a mouse model of fungal infection is a good vaccination strategy as it can protect mice against lethal doses of infection by *C. neoformans* (Mor et al., 2016).

Corroborating all the above data obtained in animal model systems that suggest antifungal activity for antibodies directed against fGlcCer, we show in this study that treatment of plants with anti-fGlcCer VHHs can reduce infection by phytopathogenic fungi such as the broad-spectrum pathogen *B. cinerea* ranked as second most important fungal plant pathogen based on scientific/economic importance (Dean et al., 2012). A comparable reduction of disease symptoms has also been reported in oat treated with defensins of *Nicotiana alata* and subsequently inoculated with *Puccinia coronata* (Dracatos et al., 2014).

For the development of a spray-on biological crop protection product it is most important that anti-fGlcCer VHHs are non-toxic, can be cost-effectively produced on industrial scale, can be stably formulated, and can exert their antifungal activity after being sprayed on the crop of interest and exposed to dry conditions for a certain period of time. The favorable safety profile of VHHs is a consequence of both their specificity and proteinaceous nature. Anti-fGlcCer VHHs are derived from naturally occurring antibodies and we showed that they bind with high specificity to fGlcCer. Compared to full-length monoclonal antibodies, VHHs have several advantages including their reduced size (approximately 120 amino acids), high solubility facilitating their high level recombinant production, their stability at high temperatures or mild concentrations of detergent, and their capacity to functionally refold after thermal, chemical or pressure denaturation (van der Linden et al., 1999; Dumoulin et al., 2002; Dolk et al., 2005). And yet, environmentally VHHs are supposedly easily degradable proteins compared to chemical compounds residing long in the environment. High-level secretion of VHHs by different yeasts such as *S. cerevisiae*, and *P. pastoris* has been described (Gorlani et al., 2012; De Meyer et al., 2014). VHH’s potentials has led to their development as therapeutics, in diagnostics, in affinity chromatography, as co-crystallizing chaperones, in preventing protein aggregation, for tuning enzyme functions, and more (de Marco et al., 2011; De Meyer et al., 2014; Wang et al., 2016). Altogether, we expect that VHHs have the same potential as biological crop protection product and after completing the challenges in production and formulation constitute a novel class of biological crop protection products. VHHs can be generated against virtually any target and VHHs may be developed as biological fungicides, insecticides, and potentially more.

## Author Contributions

BD, PV, CV, IV, MD, JV, MP, BC, and KT conceived and designed the experiments. BD, PV, CV, and IV performed the experiments. BD, PV, CV, IV, MD, and JV analyzed the experiments. BD, PV, IV, BC, and KT drafted the manuscript.

## Conflict of Interest Statement

The results of this work are subject to International Patent Applications published as WO 2014/177595 A1 and WO 2014/191146 A1. The reviewer JW and handling Editor declared their shared affiliation, and the handling Editor states that the process nevertheless met the standards of a fair and objective review.

## Supplementary Material

**FIGURE S1 |** Interaction of VHHs with fGlcCer. Optical density values (absorbance at 405 nm) represent the interaction of 10-fold diluted crude VHH-containing periplasmic extracts to 10 μg coated GlcCer from *Pleurotus citrinopileatus*.

**FIGURE S2 |** Interaction of VHHs with fGlcCer. Optical density values (absorbance at 405 nm) represent the interaction of 1 μg/ml purified VHH to coated GlcCer from porcine or soybean and was evaluated in an ELISA-based binding assay.
