# Supplementary figures and images for: Fungal Glucosylceramide-Specific Camelid Single Domain Antibodies Are Characterized by Broad Spectrum Antifungal Activity
Source: Front Microbiol. 2017 Jun 14;8:1059. doi: 10.3389/fmicb.2017.01059 (PMC5469901; doi:10.3389/fmicb.2017.01059)

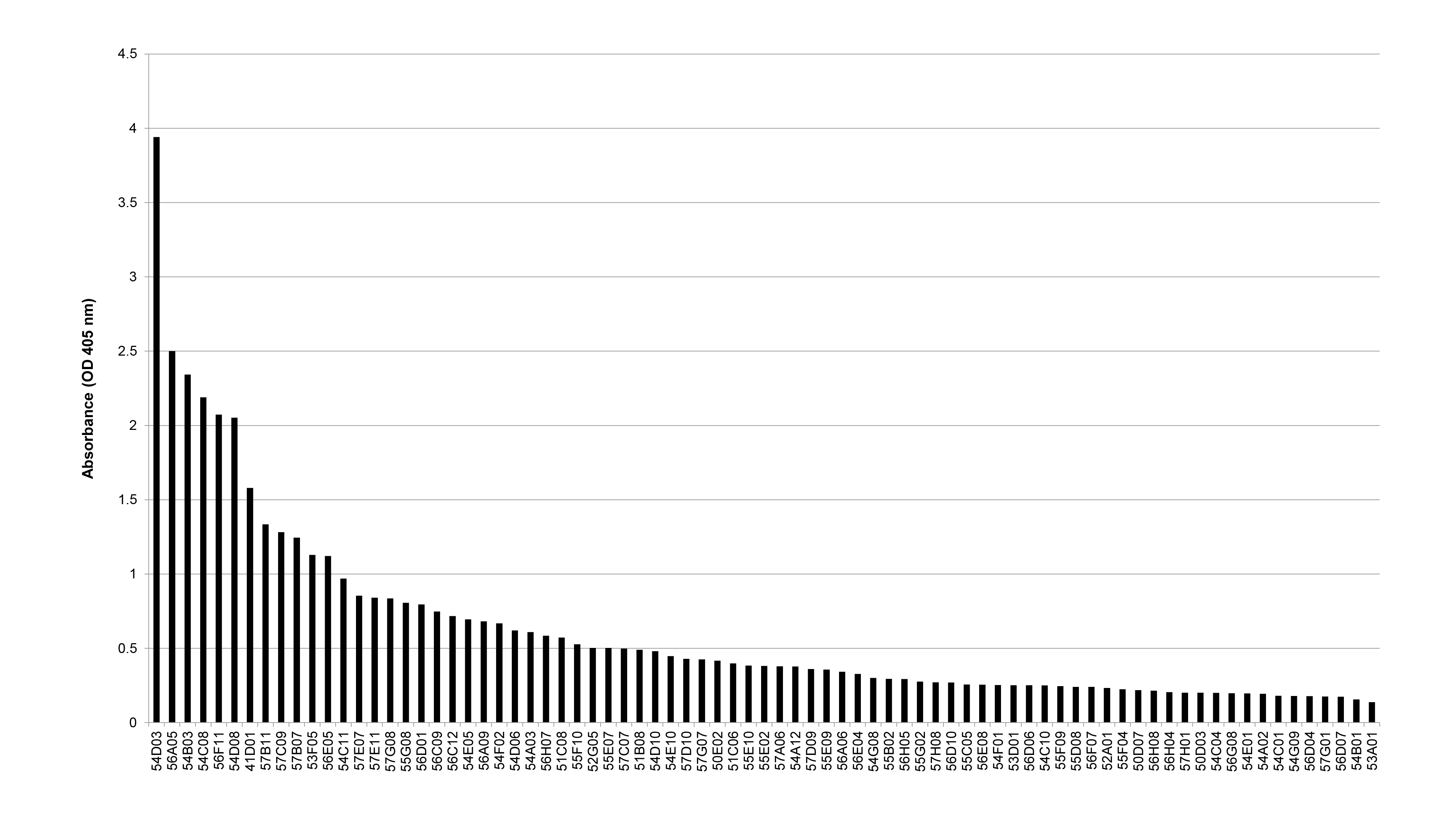

Supplement: Supplementary file 1 [file Image_1.TIF]

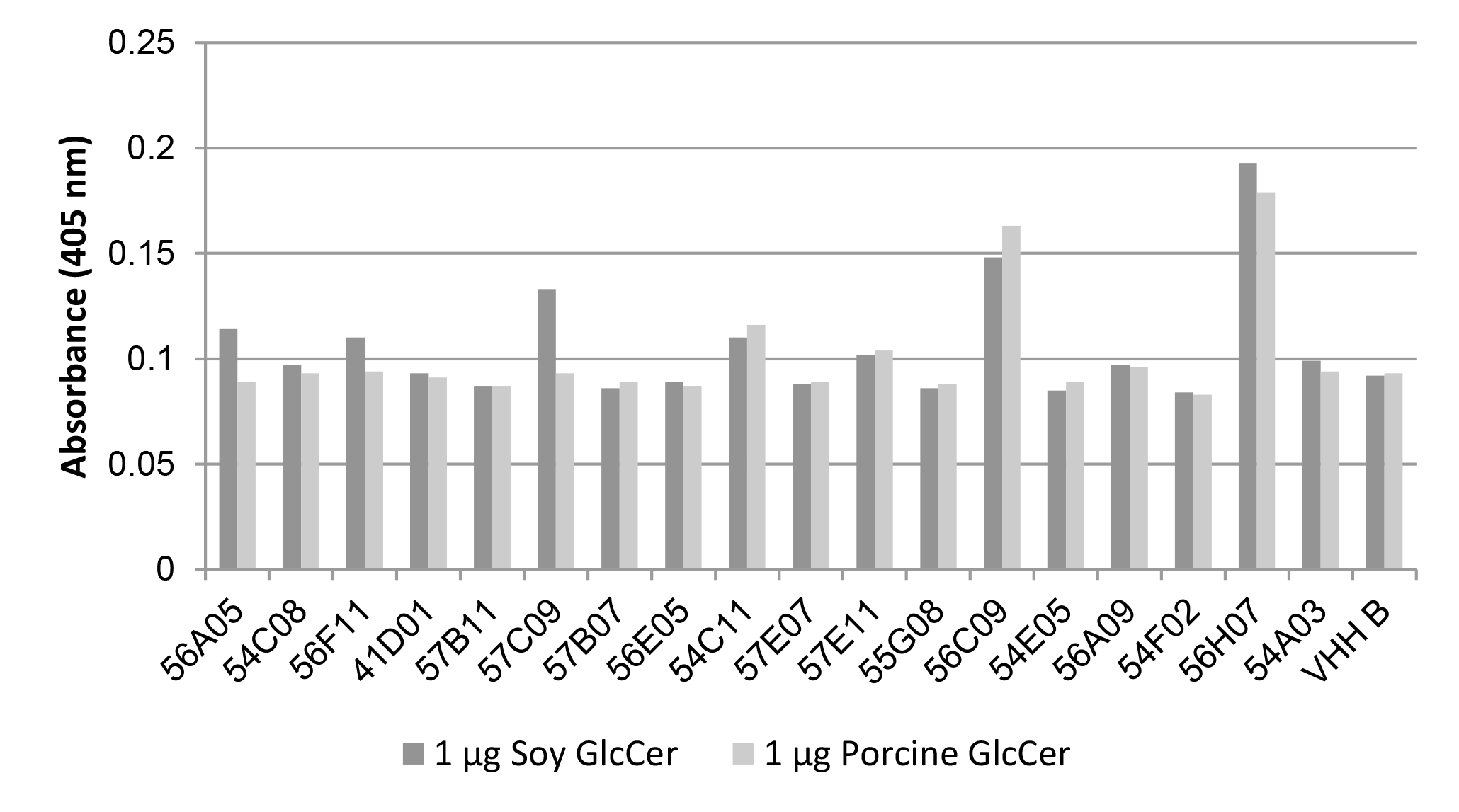

Supplement: Supplementary file 2 [file Image_2.TIF]
